# Alterations in Glutathione Levels and Apoptotic Regulators Are Associated with Acquisition of Arsenic Trioxide Resistance in Multiple Myeloma

**DOI:** 10.1371/journal.pone.0052662

**Published:** 2012-12-21

**Authors:** Shannon M. Matulis, Alejo A. Morales, Lucy Yehiayan, Kelvin P. Lee, Yong Cai, Lawrence H. Boise

**Affiliations:** 1 Departments of Hematology and Medical Oncology and Cell Biology, Emory University School of Medicine and Winship Cancer Institute, Atlanta, Georgia, United States of America; 2 Department of Microbiology and Immunology, University of Miami Miller School of Medicine, Miami, Florida, United States of America; 3 Department of Chemistry and Biochemistry, Florida International University, Miami, Florida, United States of America; 4 Departments of Immunology and Medicine, Roswell Park Cancer Institute, Buffalo, New York, United States of America; The University of Texas MD Anderson Cancer Center, United States of America

## Abstract

Arsenic trioxide (ATO) has been tested in relapsed/refractory multiple myeloma with limited success. In order to better understand drug mechanism and resistance pathways in myeloma we generated an ATO-resistant cell line, 8226/S-ATOR05, with an IC50 that is 2–3-fold higher than control cell lines and significantly higher than clinically achievable concentrations. Interestingly we found two parallel pathways governing resistance to ATO in 8226/S-ATOR05, and the relevance of these pathways appears to be linked to the concentration of ATO used. We found changes in the expression of Bcl-2 family proteins Bfl-1 and Noxa as well as an increase in cellular glutathione (GSH) levels. At low, clinically achievable concentrations, resistance was primarily associated with an increase in expression of the anti-apoptotic protein Bfl-1 and a decrease in expression of the pro-apoptotic protein Noxa. However, as the concentration of ATO increased, elevated levels of intracellular GSH in 8226/S-ATOR05 became the primary mechanism of ATO resistance. Removal of arsenic selection resulted in a loss of the resistance phenotype, with cells becoming sensitive to high concentrations of ATO within 7 days following drug removal, indicating changes associated with high level resistance (elevated GSH) are dependent upon the presence of arsenic. Conversely, not until 50 days without arsenic did cells once again become sensitive to clinically relevant doses of ATO, coinciding with a decrease in the expression of Bfl-1. In addition we found cross-resistance to melphalan and doxorubicin in 8226/S-ATOR05, suggesting ATO-resistance pathways may also be involved in resistance to other chemotherapeutic agents used in the treatment of multiple myeloma.

## Introduction

Arsenic is a naturally occurring metalloid responsible for water and crop contamination in several countries worldwide, giving rise to a significant toxicological risk [1]. Paradoxically, arsenic is also a highly effective therapeutic agent, used in several traditional Chinese medical treatments [2]. Arsenic trioxide (As_2_O_3_, ATO) is an inorganic arsenical recognized in the last 20 years for its chemotherapeutic value. In 1990s, Chinese investigators published the results of a clinical trial that revealed ATO to have significant activity in acute promyelocytic leukemia (APL) [1]. The effectiveness of ATO against APL hinges on the binding of ATO to cysteines in the PML portion of the PML-RARα fusion protein, which is present in greater than 95% of all patients diagnosed with APL [3]. This binding event leads to the degradation of the PML-RARα fusion protein, resulting in terminal differentiation and/or the induction of apoptosis [4]. In the years following the Chinese trial, several studies conducted in the United States confirmed the efficacy of treating APL with ATO and also raised the possibility of ATO use in other cancers [5].

One such malignancy since investigated is multiple myeloma (MM) [6], characterized by uncontrolled growth of antibody secreting plasma cells. At clinically achievable concentrations, ATO has been shown to induce growth arrest and apoptosis in malignant plasma cells isolated from MM patients as well as established MM cell lines [7]–[10]. Additionally, ATO exhibited modest activity against MM both as a single agent and in combination with ascorbic acid and +/− dexamethasone or bortezomib in Phase I/II clinical trails [11]–[15].

The mechanism of action whereby arsenic trioxide exerts its anti-myeloma activity has been shown to be multimodal in nature. Research studies conducted in our laboratory and by others have implicated intracellular thiol depletion, increased production of reactive oxygen species, and induction of the intrinsic apoptotic cascade as key events in ATO-induced apoptosis [9] [16]–[22]. However the molecular mechanisms that control these changes remain unclear. Therefore to gain a better understanding of how arsenic kills myeloma cells we have investigated the mechanisms associated with acquired ATO resistance. Here we present data on 8226/S-ATOR05, an arsenic trioxide resistant multiple myeloma cell line. Using gene expression profiling data, as well as previously published information on arsenic-induced pathways to guide our investigation, we identified two main pathways involved in ATO resistance in the 8226/S myeloma cell line. Our results indicate the involvement of the Bcl-2 family of proteins in controlling apoptosis at low, clinically relevant concentrations while the glutathione homeostasis pathway is important for continued viability and proliferation at high concentrations.

## Materials and Methods

### Cell Line

RPMI-8226 (8226/S) cells were purchased from American Type Culture Collection (ATCC, Manassas, VA). Cells were cultured in RPMI1640 medium, supplemented with 10% heat inactivated fetal bovine serum, 5 mM HEPES, 2 mmol/L of L-glutamine, 100 µg/mL of streptomycin, and 100 units/mL of penicillin (all purchased from Cellgro, Mediatech, Herndon, VA). Cells were maintained at 37°C in a humidified atmosphere containing 5% CO_2_.

### Generation of Arsenic Trioxide Resistant Cells

8226/S cells were initially treated with 50 nM of arsenic trioxide (ATO) and allowed to reach 50% viability before the concentration was increased. Thereafter the concentration was gradually increased each week reaching a final concentration of 1 µM ATO. 8226/S cells grown along side the resistant cell line in the absence of drug (8226-CR) and the parental cell line were used as controls.

### Reagents

Arsenic Trioxide was provided by Cell Therapeutics Inc (Seattle, WA). S-dimethylarsino-glutathione (SGLU, Darinaparsin, DAR) was provided by Ziopharm Oncology. Melphalan and Doxorubicin were purchased from Sigma-Aldrich. Bortezomib (Velcade®) was provided by Millennium Pharmaceuticals (Cambridge, MA). ABT-737 was provided by Abbott Laboratories (Abbott Park, IL). Metaphosphoric acid (MPA) used in the GSH assay was purchased from Sigma-Aldrich.

### Antibodies

Primary antibodies used are as follows: rabbit anti-HO-1 polyclonal antibody (pAb) (Santa Cruz Biotechnology, Santa Cruz, CA), rabbit anti-Nrf2 pAb (Santa Cruz Biotechnology), rabbit anti-Keap1 pAb (Proteintech Group Inc., Chicago, IL), mouse anti-NQO1 monoclonal antibody (mAb) (Cell Signaling, Danvers, MA), rabbit anti-actin pAb (Sigma-Aldrich), mouse anti-Noxa mAb (Abcam, Cambridge, MA), rabbit anti-Puma pAb (Cell Signaling), rabbit anti-Bim pAb (Chemicon International Inc, Temecula, CA), rabbit anti-Mcl-1 pAb (Stressgen Biotechnologies Corporation, Victoria, BC, Canada), rabbit anti-Bcl-x_L_ pAb (13.6) [23], and rabbit anti-Bfl-1 pAb kindly provided by Dr. Borst, The Netherland Cancer Institute. The following secondary antibodies were used: ECL Rabbit or Mouse IgG, HRP-linked Whole Ab (from donkey) (GE Heatlhcare, Piscataway, NJ), and the anti-mouse IgG1-HRP conjugate (Santa Cruz Biotechnology).

### Cell Death Determination

Cell death was measured using Annexin V-Fluorescein Isothiocyanate (FITC) (Biovision, Palo Alto, CA) and PI as previously described [19].

### Cellular Assays

ATO was removed from the culture medium of resistant cells 24 h prior to the set up of all experiments. Cells were incubated at 2.5×10^5^ cells/mL in supplemented RMPI1640 media as previously described [21] and treated with the indicated concentrations of ATO, SGLU, melphalan, doxorubicin, bortezomib, or ABT-737 for 24 h unless otherwise indicated and cell death was determined by Annexin V-FITC/PI staining, and where indicated cell pellets were frozen for protein analysis by Western blot. Cells used for the determination of resistance sustainability were cultured in the absence of arsenic for the indicated time periods before being treated with drug or collected for GSH determination.

### Western Blot Analysis

Western Blot analysis was performed using standard techniques as previously described [21].

### Total Arsenic Determination

Intracellular elemental arsenic concentrations were determined as previously described [24].

### Intracellular Glutathione Assay

GSH concentration was determined using the Glutathione Assay Kit (354102) from Calbiochem per manufacturer’s instructions. In order to relate GSH concentration to overall protein level, BCA protein assay (Pierce) was performed along side each GSH assay.

### Gene Expression Profiling

Total RNA was isolated from 8226/S, 8226/S-CR, and 8226/S-ATOR05 cells using the RNeasy® Mini Kit (Qiagen, Valencia, CA) and the hybridization and initial data analysis performed by Expression Analysis Inc. (Durham, NC). Total RNA quality was confirmed using an Agilent 2100 Bioanalyzer and cRNA was generated for probing Affymetrix Hu133 2.0 Plus Chips containing over 50,000 (54,675 including controls) probe sets. Affymetrix GCOS software was used with statistical algorithms to determine a quantitative value (signal intensity) and a qualitative value [present (P) or absent (A) calls] for each transcript on the array. Signal intensities for each cell line were considered only if at least one present call, as qualitative value, was reported for any time point and at least one signal was higher than 100. Data were analyzed using Microsoft Excel and the bioinformatic programs Cluster and TreeView (Eisen Laboratory, University of California, CA) as previously described [21] [25].

### Real-Time PCR

Total RNA was extracted from parental, CR, and resistant cells at the time points indicated using the RNeasy Mini Kit (Qiagen). One microgram of total RNA, MuLV Reverse Transcriptase, and random hexamer primers from the GeneAmp RNA PCR Kit (Applied Biosystems) were used to generate cDNA. Subsequent cDNA was amplified using the 20× human Noxa mix (PMAIP1, Hs00560402_m1) or Bfl-1 mix (BCL2A1, Hs00187845_m1) and the TaqMan Gene Expression Assay (Applied Biosystems) on the 7700 Sequence Detection System following the manufacturer’s protocol. TaqMan human GAPDH (402869) was used as an internal control and target mRNA expression was calculated relative to untreated parental control, using GAPDH to normalize RNA expression.

## Results

### 8226/S Cell Line can Acquire Resistance to Arsenic Trioxide

**Figure 1:**
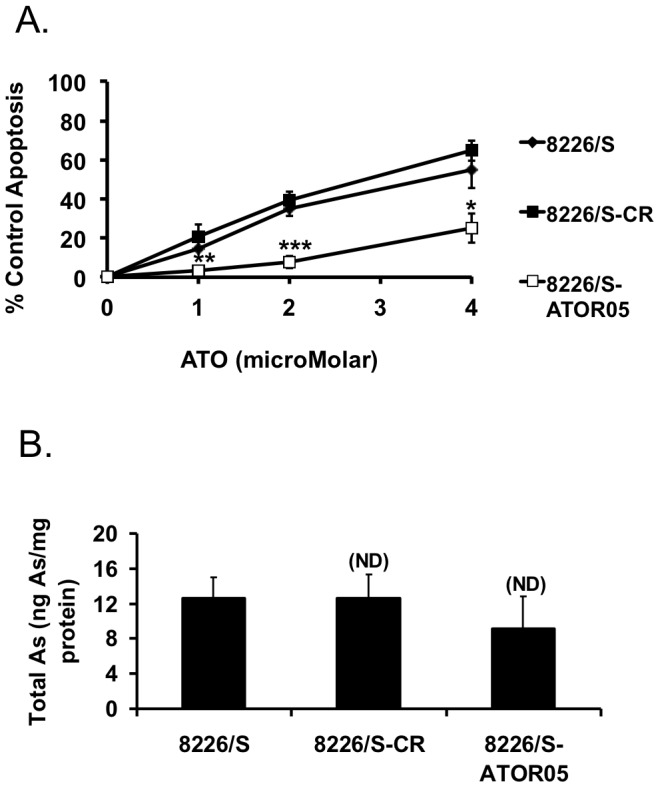
8226/S-ATOR05 cell line is resistant to ATO and resistance is not due to changes in uptake. (**A**) 8226/S, 8226/S-CR, 8226/S-ATOR05 cell lines were treated with the indicated concentrations of ATO for 24 h and apoptosis was assessed by flow cytometry using Annexin V-FITC and PI staining and graphed as percent of control Annexin V positive cells versus drug concentration. (**B**) 8226/S, 8226/S-CR, 8226/S-ATOR05 cells were treated with 2 µM ATO for 24 h and then collected for intracellular arsenic determination. The data are presented as the mean ± SD of at least 3 independent experiments. Student’s *t*-test was used to compare differences between 8226/S and 8226-CR and 8226/S-ATOR05 cells, with 95% confidence intervals. *, P<0.05; **, P<0.01; ***, P<0.001.

In order to better understand the mechanisms governing the anti-myeloma activity of ATO, we generated an ATO resistant cell line. We attempted to generate four arsenic resistant lines, using KMS11, MM.1s, U266, and 8226/S myeloma cell lines, however we were only able to successfully produce a resistant cell line in 8226/S (8226/S-ATOR05). To control for changes that are the result of the time required for the selection, we cultured cells in the absence of arsenic parallel to the selected cells. These cells are referred to as control resistant (CR) cells. Figure 1A represents a 24 hour dose curve with 8226/S, 8226/S-CR, and 8226/S-ATOR05 cell lines. The parental and CR cell lines exhibit a similar apoptotic dose response with IC50s of 3.46 µM and 2.93 µM respectively. In contrast, the IC50 for 8226/S-ATOR05 is 2–3-fold higher than the control cell lines at 8.85 µM, representing a statistically significant resistance to ATO that is well beyond clinically achievable concentrations.

In order to verify that arsenic resistance in 8226/S-ATOR05 is not simply due to lack of arsenic transport, we performed arsenic uptake assays. Control and resistant cells were treated with 2 µM ATO for 24 hours and the level of elemental arsenic present in each cell line was determined. We found no significant difference in the intracellular concentration of arsenic between the parental, CR, and resistant cell lines (Fig. 1B).

Additionally we analyzed gene expression profiling data for these cells to determine if arsenic resistance in 8226/S-ATOR05 was associated with changes in the expression of any ABC transporters (Table 1). We were unable to correlate any changes in pump expression to arsenic resistance, indicating resistance is not due to an increase in the expression of drug efflux pumps. Together these data suggest that ATO resistance in 8226/S-ATOR05 cells is due to biochemical changes in arsenic response pathways and not to changes in uptake or efflux of ATO or its metabolites.

**Table 1:** ABC genes relative expression.

ABC Genes	Alias	8226/S	8226/S-CR	8226/S-ATOR05	8226/S-ATOR05	8226/S-ATOR05
					NO ATO 23 d	NO ATO 50 d
ABCA1	ABC1	1.00	1.35	1.21	1.15	1.37
**ABCA2**	**ABC2**	1.00	0.74	0.99	0.98	0.94
ABCA7		1.00	0.92	0.82	0.73	0.68
ABCB6	MTABC3	1.00	0.75	1.04	1.73	0.96
ABCB7	ABC7	1.00	1.46	1.19	0.99	1.09
ABCB8	MABC1	1.00	1.35	1.40	1.54	1.77
ABCB10	MTABC2	1.00	1.23	0.94	0.87	0.84
**ABCC1**	**MRP1**	1.00	1.19	1.45	1.66	1.48
ABCC4	MRP4	1.00	0.88	0.99	0.80	0.93
ABCC5	MRP5	1.00	1.04	0.76	0.65	0.57
ABCC10	MRP7	1.00	0.97	1.00	1.05	0.94
ABCD1	ALD	1.00	1.85	3.20	4.62	3.80
ABCD3	PXMP1,PMP70	1.00	1.03	0.79	0.73	0.90
ABCD4	PMP69, P70R	1.00	0.77	0.94	1.06	1.07
ABCE1	OABP, RNS4I	1.00	1.05	1.04	0.99	0.94
ABCF1	ABC50	1.00	1.05	1.15	1.13	1.25
ABCF2		1.00	0.93	1.14	0.96	1.15
ABCF3		1.00	1.24	1.07	1.18	1.31
ABCG1	ABC8, White	1.00	0.73	1.17	1.19	0.68
**ABCG2**	**ABCP, MXR, BCRP**	1.00	0.89	0.55	0.64	0.60

Genes associated to drug resistance are in bold. Genes that were not expressed are not listed.

### The Antioxidant Response Remains Unchanged While the Apoptotic Response is Altered in Cells that have Acquired ATO Resistance

**Figure 2:**
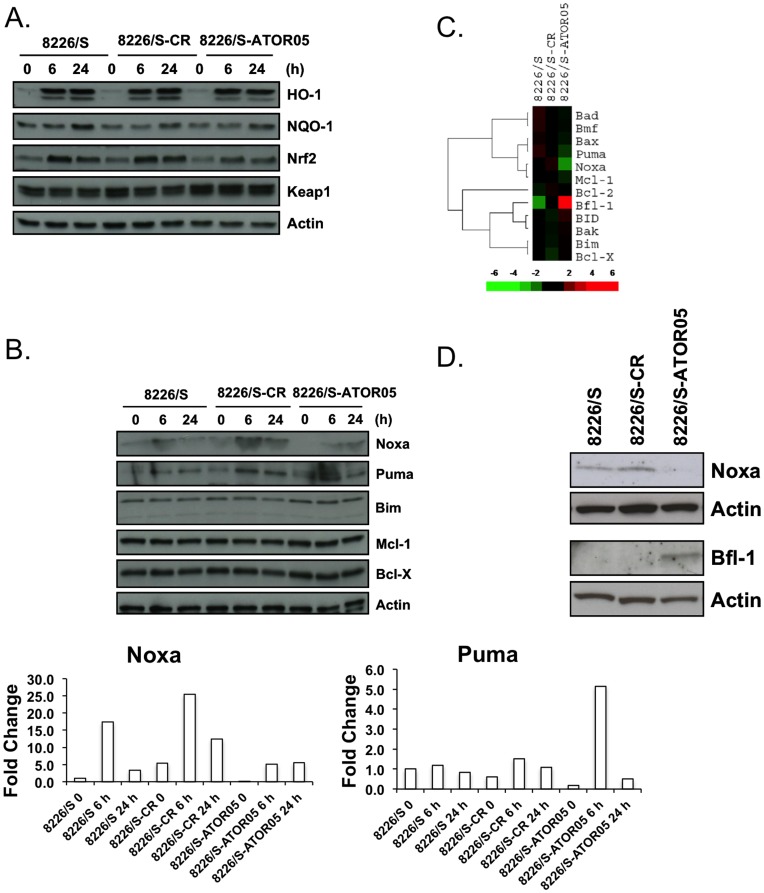
The antioxidant response is unchanged in 8226/S-ATOR05 cells while the expression of Bcl-2 family members is altered. 8226/S, 8226/S-CR, 8226/S-ATOR05 cells were treated with 2 µM ATO for 0, 6, or 24 h, protein expression was determined by Western blot and membranes were probed with antibodies against proteins involved in the (**A**) antioxidant response or (**B**) the Bcl-2 family. (**C**) A heatmap of Bcl-2 genes expressed in 8226/S cells. The scale represents the change relative to the median expression for each gene. (**D**) Protein expression of Noxa and Bfl-1 was determined by Western blot in 8226/S, 8226/S-CR, 8226/S-ATOR05 control cells. Actin expression demonstrates protein loading. Quantitation of changes in Noxa and Puma are provided. The data are normalized to actin and presented as fold change relative to untreated parental cells.

We next turned our attention to changes in previously documented responses to ATO treatment – the antioxidant response and the apoptotic response. We have previously documented the activities of the antioxidant response through the stabilization of the transcription factor Nrf2 following ATO treatment of myeloma cell lines [25]. Nrf2 regulates the expression of many genes that protect cells from oxidative damage, including heme oxygenase-1 (HO-1) and NAD(P)H dehydrogenase quinone 1 (NQO1) [26]. Therefore we determined if the anti-oxidant response was altered in ATOR05 cell by measuring expression of Nrf2 and its targets, NQO1 and HO-1. Surprisingly we did not see any effect on the basal expression of any of these proteins or the Nrf2 regulator KEAP1 (Figure 2A). Additionally, following ATO addition, the anti-oxidant response was similar in all three cell lines as Nrf2, NQO1 and HO-1 were all induced (Figure 2A). While these data demonstrate that the ATOR05 cells respond to ATO treatment, they also suggest that the Nrf2-dependent anti-oxidant response plays little role in the activity of ATO in myeloma cells.

Changes in the expression pattern of pro-apoptotic BH3-only proteins are also part of the response to ATO treatment of MM cells. We, and others, previously demonstrated the importance of BH3-only proteins Noxa, Bim, and Bmf in arsenic-induced apoptosis [21] [27]. We did not see significant differences in the baseline levels of the anti-apoptotic proteins Bcl-x_L_ and Mcl-1 or the BH3-only proteins Bim and Puma. However, Noxa levels appeared lower in the ATO-resistant cells and the induction of Noxa and Puma was diminished following ATO-treatment (Figure 2B).

In order to further investigate the role of the Bcl-2 family of proteins in acquired arsenic resistance, we performed gene expression profiling on parental, CR, and resistant untreated cells (Fig. 2C). Analysis of these data revealed changes in the regulation of both pro- and anti-apoptotic genes. Consistent with what was observed in Figure 2B, mRNA levels of BH3-only proteins Noxa and Puma were down-regulated 4- and 2-fold respectively. Interestingly the anti-apoptotic Bfl-1 was up-regulated 4-fold. Western blot analysis was performed in order to confirm if these changes were also present at the protein level. As seen in Figure 2D, a decrease in the expression of Noxa and an increase in the expression of Bfl-1 are observed at the protein level in the arsenic resistant cell line 8226/S-ATOR05. While baseline Puma expression was lower in ATOR05 cells compared to parental cells, induction remained intact (Figure 2D).

### Glutathione Levels in 8226/S-ATOR05 Cells are Significantly Increased as Compared to Control Cell Lines

**Figure 3:**
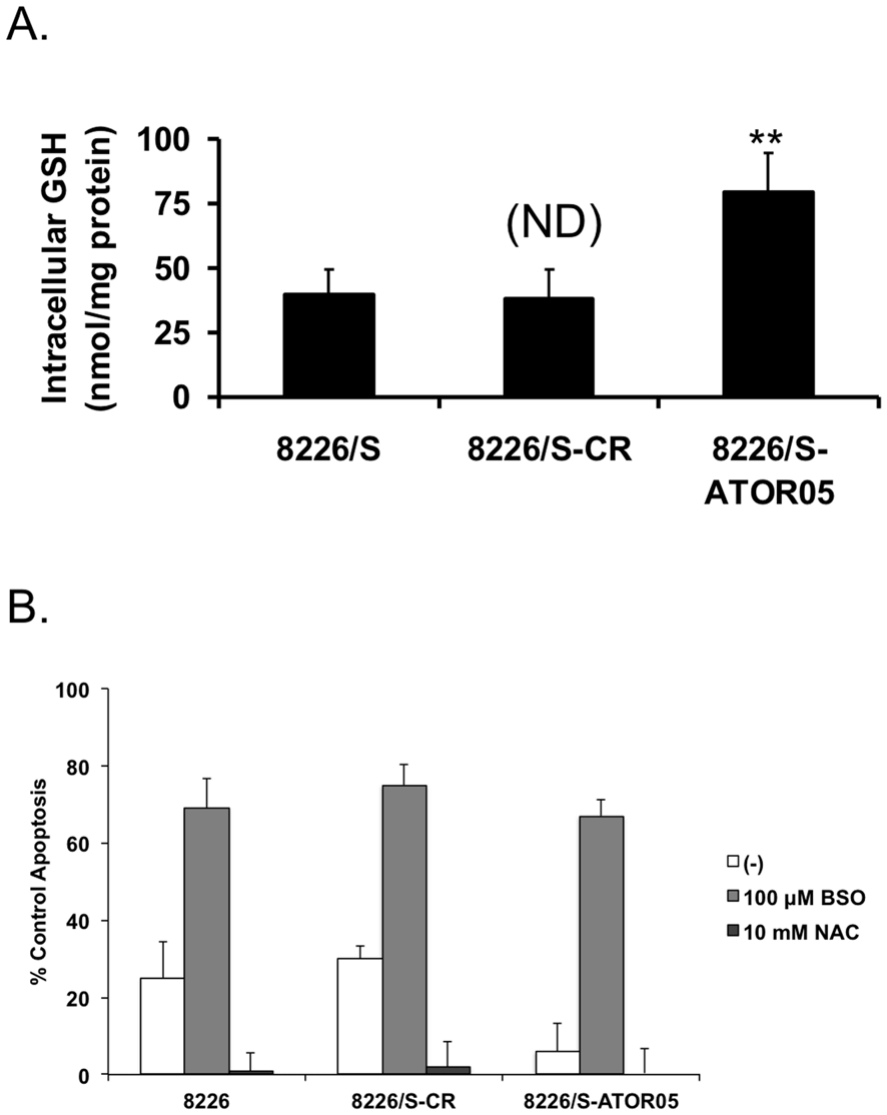
The intracellular GSH level is elevated in 8226/S-ATOR05 cells and is important for arsenic resistance. (**A**) 8226/S, 8226/S-CR, 8226/S-ATOR05 control cells were harvested and the intracellular GSH concentration was determined using the Glutathione Assay kit from Calbiochem. (**B**) 8226/S, 8226/S-CR, 8226/S-ATOR05 cells were treated with 2 µM ATO alone or in combination with either 100 µM BSO or 10 mM NAC for 24 h and apoptosis was determined using flow cytometry. Data are graphed as percent of control Annexin V positive cells. Mean ± SD of at least 3 independent experiments. **, P<0.01.

The role of glutathione (GSH) in arsenic metabolism and detoxification is well established [28], as well as the role of increased intracellular glutathione in drug resistance [29]. Therefore we next determined the level of intracellular GSH present in the resistant cell line, as compared with the controls. Data presented in Figure 3A verify a significant increase in the amount of intracellular GSH present in 8226/S-ATOR05 when compared to either 8226/S or 8226/S-CR. On average, resistant cells contained 79.59 nmol GSH/mg of protein, whereas the control and control resistant cells contained 39.45 and 37.91 nmol GSH/mg protein, respectively.

In order to determine if this increase in intracellular GSH is important for establishing resistance to ATO, we treated parental, control resistant, and resistant cells with either buthionine sulfoximine (BSO) or N-acetyl-cysteine (NAC) for 24 hrs. BSO inhibits the rate-limiting enzyme in GSH synthesis, γ-glutamate cysteine ligase, blocking production, whereas NAC increases the available intracellular cysteine thereby boosting GSH production. Increasing GSH concentration through NAC treatment allowed 8226/S and 8226/S-CR cells to survive in the presence of ATO at viabilities comparable to that of resistant cells treated with ATO alone. In contrast NAC had no effect on ATO-induced apoptosis in the ATO-resistant cells. However, treating resistant cells with ATO and BSO resulted in significant cell death, underlining the importance of glutathione in arsenic resistance.

Next we wanted to determine if the baseline GSH increase in resistant cells was due to changes in the transcription of genes involved in glutathione homeostasis. Table 2 lists the expressed genes associated with glutathione metabolism and their relative expression based on gene expression profiling. Most genes associated with GSH synthesis or utilization were either unchanged or displayed a change of less than 50% when compared to the parental line (Table 2). However four genes were upregulated by acquisition of resistance to arsenic and returned to baseline following arsenic removal that also resulted in a return to baseline GSH levels (see below). While two of these genes are involved in the use of GSH (GSTM1, MGST3), the other two genes encode proteins that could result in increased GSH levels. Esterase D (ESD) is a serine hydrolase that is involved in formaldehyde detoxification. It catalyzes the hydrolysis of S-formylglutathione to GSH and formate [30]. However it is unlikely that these cells contain significant levels of S-formylglutathione. In contrast the fourth gene that is upregulated in 8226/S-ATOR05 and returns towards baseline when the cells are removed from selection is required for *de novo* GSH synthesis. Glutathione synthetase (GSS) catalyzes the final step in GSH synthesis [31] and is 1.48-fold higher in ATOR05 cells.

**Table:** **Table 2.** Relative expression of GSH-associated genes.

Gene Descriptor	Gene Symbol	8226/S	8226/S-CR	8226/S-ATOR05	8226/S-ATOR05	8226/S-ATOR05
					NO ATO 23 d	NO ATO 50 d
Cystathionase (cystathionine gamma-lyase)	CTH	1.00	1.36	**0.49**	**0.42**	**0.70**
***Esterase D/formylglutathione hydrolase***	ESD	1.00	0.95	**1.58**	1.33	1.24
Gamma-glutamyltransferase 1	GGT1	1.00	0.82	0.96	0.93	0.96
Glutamate-cysteine ligase, catalytic subunit	GCLC	1.00	1.02	1.08	1.14	1.26
Glutamate-cysteine ligase, modifier subunit	GCLM	1.00	1.22	0.95	0.98	**1.47**
Glutathione peroxidase 1	GPX1	1.00	**1.46**	0.77	1.18	0.75
Glutathione peroxidase 4	GPX4	1.00	1.00	1.32	1.04	0.80
Glutathione peroxidase 7	GPX7	1.00	**0.50**	0.74	1.02	0.75
Glutathione reductase	GSR	1.00	0.86	1.27	**1.63**	**1.49**
Glutathione S-transferase A4	GSTA4	1.00	0.93	**1.87**	**2.17**	**1.55**
Glutathione S-transferase kappa 1	GSTK1	1.00	0.86	1.13	1.00	0.78
***Glutathione S-transferase M1***	GSTM1	1.00	0.93	**1.71**	1.31	1.00
Glutathione S-transferase M2 (muscle)	GSTM2	1.00	0.93	1.38	1.28	1.36
Glutathione S-transferase omega 1	GSTO1	1.00	0.82	1.01	0.88	0.79
Glutathione S-transferase theta 1	GSTT1	1.00	0.73	**0.71**	0.72	**0.65**
Glutathione S-transferase, C-terminal domain containing	GSTCD	1.00	1.06	0.92	1.03	1.24
***Glutathione synthetase***	GSS	1.00	0.71	**1.48**	1.37	1.12
Glutathione transferase zeta 1	GSTZ1	1.00	1.09	0.83	1.14	1.26
Hydroxyacylglutathione hydrolase	HAGH	1.00	0.98	0.56	0.86	0.67
Hydroxyacylglutathione hydrolase-like	HAGHL	1.00	1.05	1.40	1.19	0.98
Malic enzyme 1, NADP(+)-dependent, cytosolic	ME1	1.00	0.91	1.07	1.05	1.07
Malic enzyme 2, NAD(+)-dependent, mitochondrial	ME2	1.00	0.79	0.72	0.63	**0.50**
Malic enzyme 3, NADP(+)-dependent, mitochondrial	ME3	1.00	**0.60**	0.79	0.94	**1.47**
Microsomal glutathione S-transferase 1	MGST1	1.00	0.94	1.03	0.93	0.96
Microsomal glutathione S-transferase 2	MGST2	1.00	**0.68**	0.96	**1.49**	**1.48**
***Microsomal glutathione S-transferase 3***	MGST3	1.00	**0.69**	**1.62**	1.15	1.21
Serine hydroxymethyltransferase 1 (soluble)	SHMT1	1.00	1.22	1.01	1.23	1.09
Serine hydroxymethyltransferase 2 (mitochondrial)	SHMT2	1.00	1.01	0.97	0.92	0.91
Serine palmitoyltransferase, long chain base subunit 2	SPTLC2	1.00	1.07	0.91	1.34	1.36
Solute carrier family 7, member 11	SLC7A11	1.00	0.85	**0.67**	**0.56**	0.94

Expression is relative to the parental cell line 8226/S. Changes in expression greater or equal to 1.4 are highlighted in bold. Genes changes that are associated with the ATO-resistant phenotype are shown in bold and italics.

### Removal of Arsenic Selection Results in Loss of Resistance Phenotype

**Figure 4:**
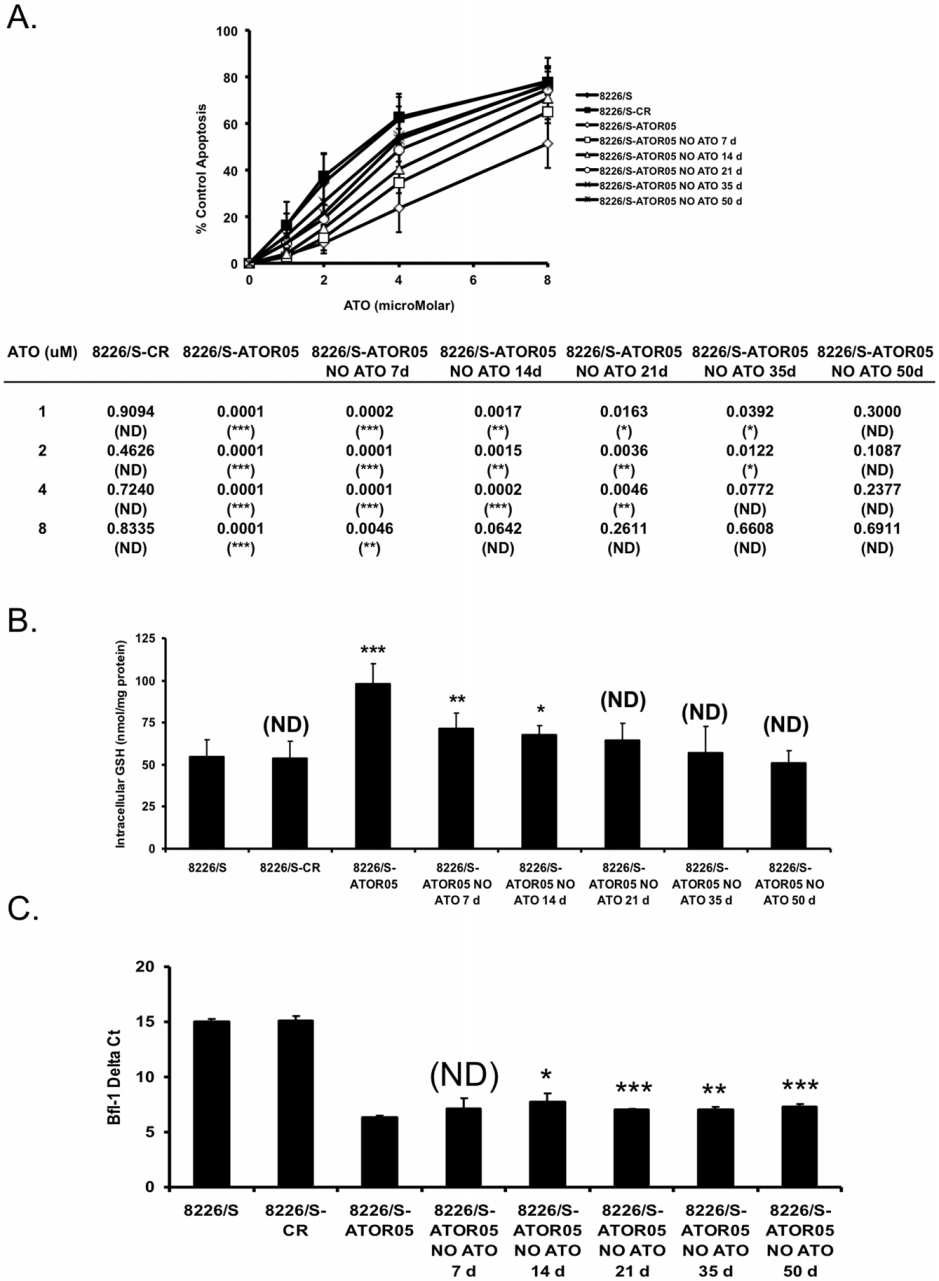
The ATO resistance phenotype is lost upon removal of arsenic. (**A**) 8226/S, 8226/S-CR, 8226/S-ATOR05 cells, as well as 8226/S-ATOR05 cells cultured in the absence of ATO for 7, 14, 21, 35, and 50 days were treated with the indicated concentrations of ATO for 24 h and apoptosis was determined by flow cytometry and graphed as percent of control Annexin V positive cells versus drug concentration. Statistical analysis compared to 8226/S are provided in the lower panel. (**B**) 8226/S, 8226/S-CR, 8226/S-ATOR05 control cells, as well as control cells from the indicated time points were harvested and the intracellular GSH concentration was determined as described in the Materials and Methods. (**C**) Real-time PCR was performed on 8226/S, 8226/S-CR, 8226/S-ATOR05 cells, as well as 8226/S-ATOR05 cells cultured in the absence of ATO for 7, 14, 21, 35, and 50 days to determine the expression patterns of Bfl-1. Data is graphed using the Delta CT, which represents the number of cycles needed to reach threshold, normalized against GAPDH. High delta Ct values represent low expression while low delta Ct values indicate high expression. The data in (B) and (C) are presented as the mean ± SD of at least 3 independent experiments. Student’s *t*-test was used to compare differences between 8226/S-ATOR05 and all other cell lines, with 95% confidence intervals. *, P<0.05; **, P<0.01; ***, P<0.001, No difference (ND).

To determine if arsenic resistance in 8226/S-ATOR05 cells is dependent upon the selective pressure of arsenic, 24 h dose curves were performed on parental, CR, and resistant cells cultured in the absence of ATO for 7, 14, 21, 35, and 50 days (Figure 4A). At the highest concentration tested (8 µM), changes in sensitivity were observed as early as 7 days after ATO withdrawal, however sensitivity to a clinically relevant concentration (2 µM) was not observed until 50 days following ATO withdrawal. These data suggest that the changes associated with high level resistance are exquisitely dependent upon the sustained presence of arsenic while resistance to clinically relevant levels of arsenic can still be observed for an extended period following removal.

In order to determine if this loss of resistance was associated with changes in GSH levels, we examined the levels of intracellular GSH in 8226/S-ATOR05 cells cultured in the absence of ATO for 7, 14, 21, 35, and 50 days (Figure 4B). The level of intracellular GSH decreased in a time dependent fashion following arsenic removal, with no significant difference in GSH concentration compared to the control cell lines by 21 days out. These data suggest that the presence of arsenic is required to maintain increased GSH production and high level resistance in the ATO resistant cells.

As presented in Figure 2, changes in the expression of Noxa and Bfl-1 may also contribute to the arsenic resistant phenotype. Given that removal of arsenic from the culture medium leads to a gradual return to sensitivity at therapeutic levels, we wanted to determine the fate of Noxa and Bfl-1 expression in this transition. Gene expression was determined using Real Time qPCR, including samples from resistant cells cultured in the absence of arsenic for 1, 7, 14, 21, 35, and 50 days. Figure 4C shows a dramatic increase in the level of Bfl-1 mRNA in 8226/S-ATOR05 cells. This expression decreases by approximately 50% as cells are maintained in arsenic-free culture medium, but it never returns to control levels. Noxa levels were lower in the ATOR5 cells, however they increased minimally after arsenic removal (not shown). Taken together these data presented suggest that a gradual decrease in GSH levels as well as a gradual decrease in Bfl-1 expression and a modest increase in Noxa levels collaborate to return ATO sensitivity to 8226/S-ATOR05 cells in the absence of arsenic selectivity.

**Figure 5:**
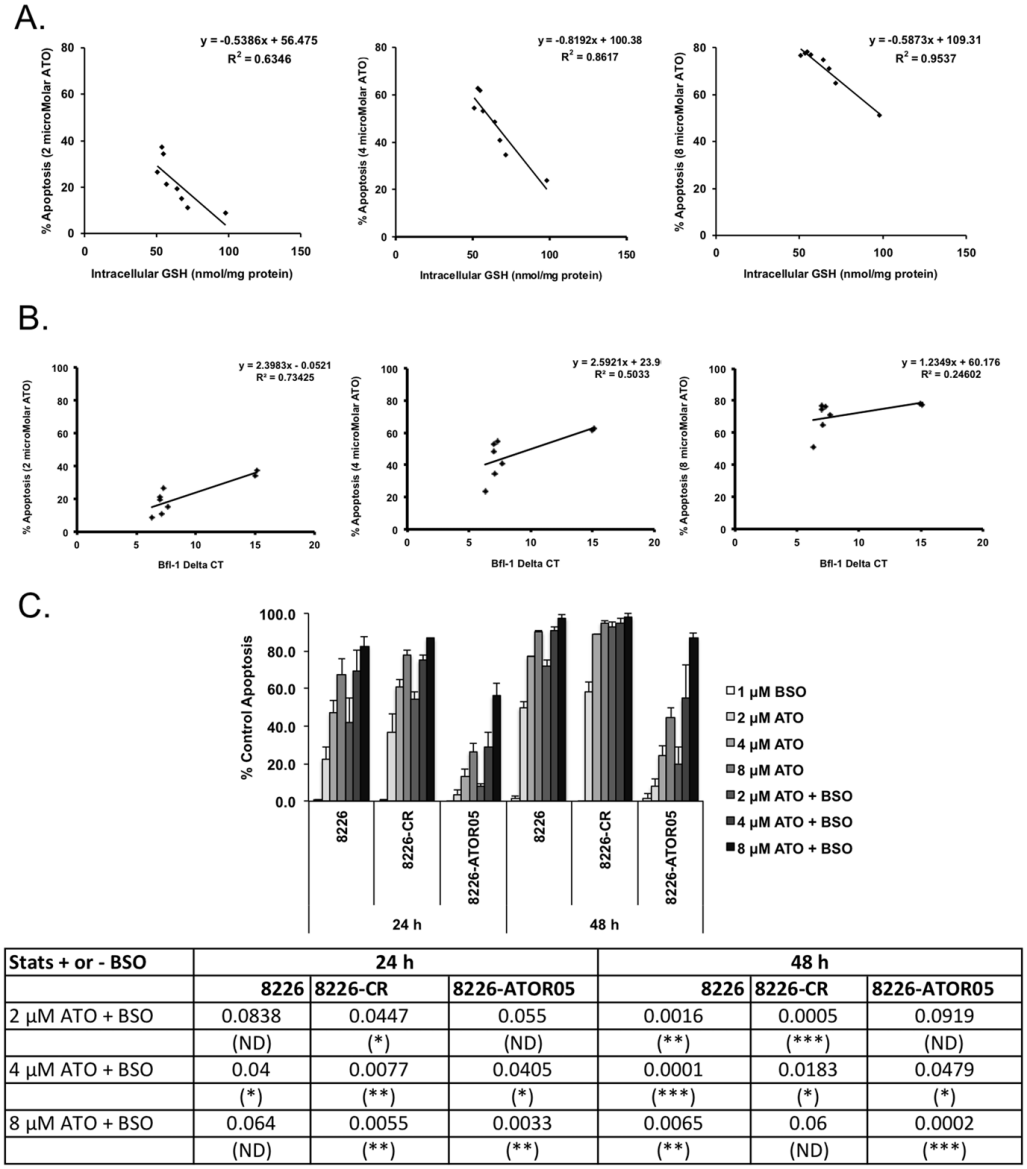
GSH inversely correlates with response to high concentrations of ATO while Bfl-1 expression inversely correlates with the response to clinically relevant ATO concentrations. GSH levels (**A**) or Bfl-1 expression (**B**) from Figure 4 was correlated with apoptosis data from Figure 4. Pearson coefficient data are presented as a determinant of the strength of the correlation. (**C**) Cells were treated with the indicated concentrations of ATO in the presence or absence of BSO (1 µM) and apoptosis determined at 24 and 48 h. The data are presented as the mean ± SD of at least 3 independent experiments. The lower panel provides the statistical analyses of the data (student’s t test).

These data suggest a possible relationship between the level of intracellular GSH, the expression of Bfl-1/Noxa and arsenic sensitivity. To test if a correlation exists, we compared intracellular GSH concentrations (Figure 5A) and Bfl-1 expression (Figure 5B) observed during the 50 day time course following ATO removal to the sensitivity to arsenic at each time point. This analysis revealed an inverse correlation between intracellular GSH level or Bfl-1 expression and arsenic sensitivity. No correlation was observed with Noxa expression (not shown). Interestingly these correlations were stronger at different concentrations of ATO. In the case of GSH, this inverse correlation is nearly linear at 8 µM ATO, while at the more clinically relevant concentration of 2 µM, the association exists, however it is not as strong (R^2^ = 0.9537, 0.6346 respectively). In contrast a correlation exists at the lower concentration of ATO and Bfl-1 expression (R^2^ = 0.73425) while no correlation exists at the highest concentration (R^2^ = 0.24602). Taken together with the temporal differences observed (intracellular GSH returns to baseline by 21 days while resistance persists for an additional 29 days and Bfl-1 levels persist) these data suggest that at high ATO concentrations, GSH is the primary contributor to acquired resistance, while at clinically relevant concentrations a change in the apoptotic threshold associated with increased Bfl-1 also contributed to the response.

To further test the role of GSH in acquired ATO resistance, we tested the effects of GSH depletion on both high and low concentrations of ATO in the ATOR05 and control lines. Since depletion of GSH with 100 µM BSO will sensitize both the parental ATOR05 cells to 2 µM ATO (Figure 3B), we initially titrated the BSO to the lowest concentration that had a significant effect in the parental cells treated with 2 µM ATO. This allowed us to observe differences in the effects of BSO on increasing concentrations of ATO. We found that we could use as little as 1 µM BSO to sensitize the parental cells to 2 µM ATO while having little effect in ATOR05 (Figure 5C and data not shown). In the parental cell line the addition of BSO had a significant impact on ATO-induced apoptosis at all concentrations of ATO tested at 48 h (Figure 5C). Similar results were observed for the CR line however the effect of BSO was not significant at 8 µM (P = 0.06). This is likely due to the fact that almost all the cells are already dead with ATO alone at this concentration. In contrast BSO has no effect on ATO-induced apoptosis at 2 µM and is marginally significant at 4 µM (p = 0.0479) in the ATO-resistant line. However BSO has a highly significant (p = 0.0002) effect in these cells when used in combination with 8 µM ATO. Together these data support the conclusion that the primary role for increased GSH is to protect ATO-resistant cells from high concentrations of ATO.

### ATO Resistant Cell Line 8226/S-ATOR05 also Displays Resistance to Melphalan and Doxorubicin

**Figure 6:**
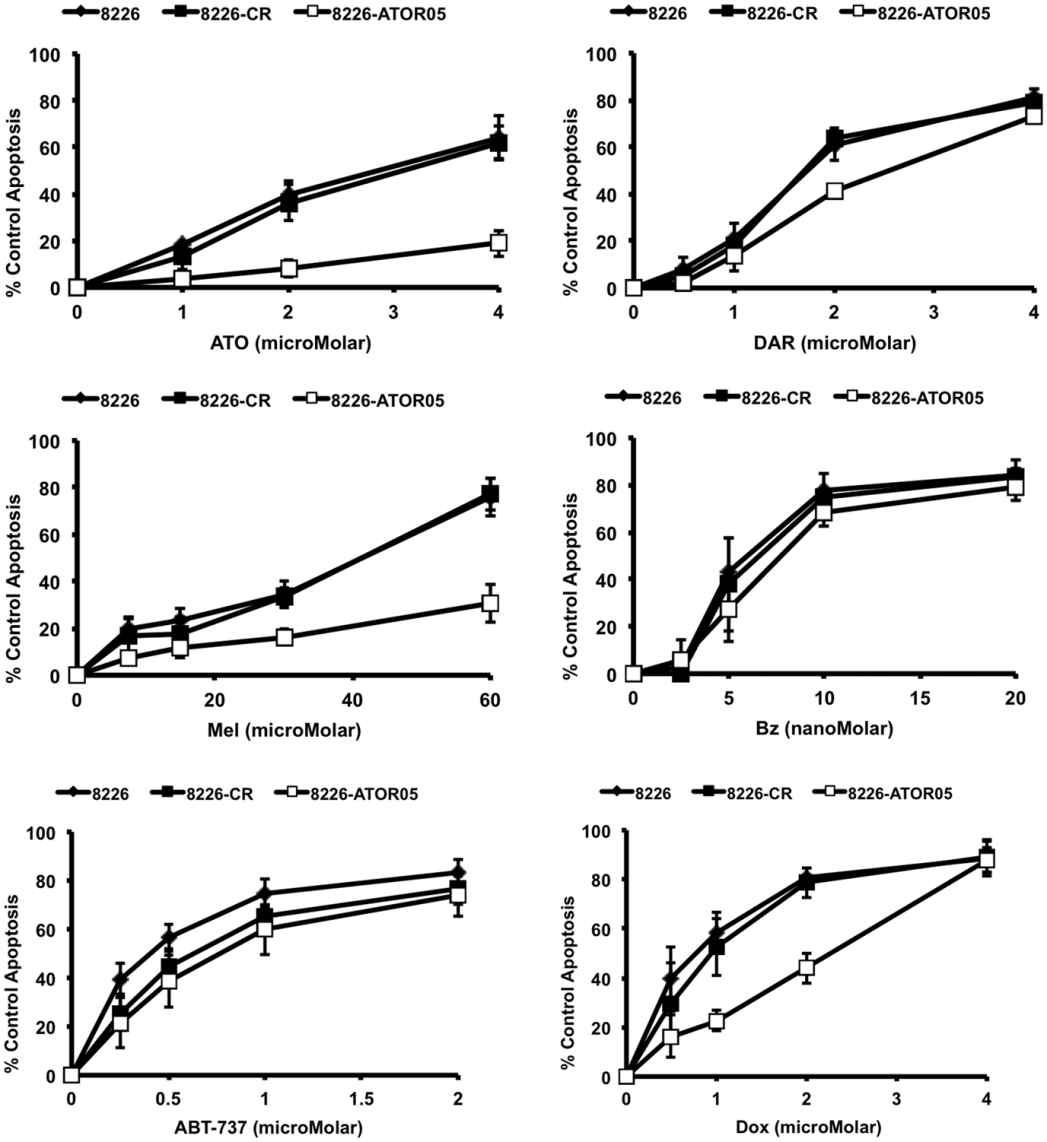
ATO resistant cells are also resistant to melphalan and doxorubicin, but remain sensitive to darinaparsin, bortezomib and ABT-737. 8226/S, 8226/S-CR, and 8226/S-ATOR05 cells were treated with the indicated concentrations of ATO, DAR, Mel, Bz, ABT-737 or Dox for 24 h and apoptosis was assessed by flow cytometry and graphed as percent of control Annexin V positive cells vs. drug concentration. The data are presented as the mean ± SD of at least 3 independent experiments.

In order to determine if acquisition of ATO resistance also confers resistance to other commonly used or novel chemotherapeutic agents, we tested the sensitivity of parental, CR, and ATO resistant cell lines to darinaparsin (S-dimethylarsino-glutathione, SGLU), melphalan, bortezomib, ABT-737, and doxorubicin. Figure 6A presents 24 h concentration curves with each drug, as well as ATO. ATO resistant cells remain sensitive to the organic arsenical darinaparsin (DAR), the proteasome inhibitor bortezomib, and the Bcl-2/x_L_ inhibitor ABT-737, but show cross-resistance to melphalan and doxorubicin. The concentration curves of ATO and melphalan-treated resistant cells are very similar, maintaining resistance at all concentrations investigated. However, doxorubicin-treated ATO-resistant cells display resistance at low concentrations but become as sensitive as control cells at the highest concentration tested. These data suggest that the mechanism by which myeloma cells respond to melphalan is similar to that of ATO, while the mechanism for doxorubicin maybe more distantly related.

**Figure 7:**
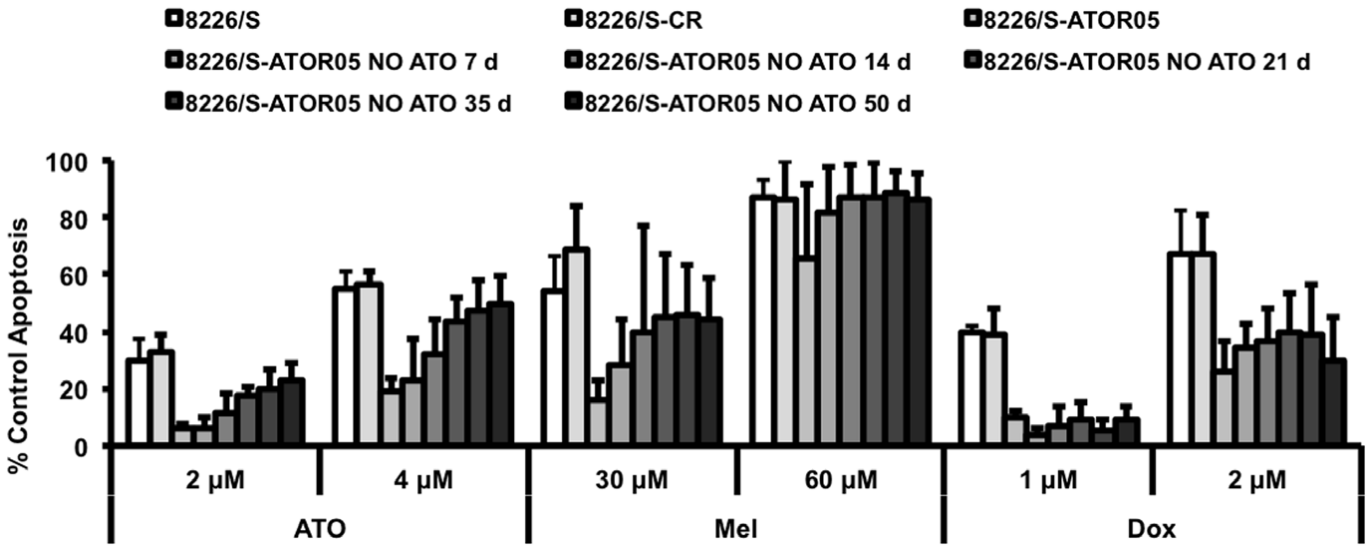
ATO and Melphalan resistance pathways overlap while Doxorubicin resistance pathway(s) may be related. 8226/S, 8226/S-CR, 8226/S-ATOR05 cells, as well as 8226/S-ATOR05 cells cultured in the absence of ATO for 7, 14, 21, 35, and 50 days were treated with the indicated concentrations of ATO, Mel, or Dox for 24 h and apoptosis was determined by flow cytometry and graphed as percent of control Annexin V positive cells versus drug concentration. The data are presented as the mean ± SD of at least 3 independent experiments.

In order to determine if the resistance of 8226/S-ATOR05 to melphalan and doxorubicin is dependent on the ATO resistant phenotype, resistant cells were cultured in the absence of ATO for 7, 14, 21, 35, or 50 days, then treated with two concentrations of each drug for 24 h (Figure 7). Cells treated with 30 µM melphalan behaved in a manner similar to that of cells treated with ATO, regaining sensitivity to drug as time from arsenic removal increased, however treatment with 60 µM melphalan resulted in significant apoptosis as early as 7 days out. These data indicate arsenic-resistance mechanisms maybe overcome with high dose melphalan. Interestingly, all variations of 8226-ATOR05 treated with doxorubicin maintained resistance at both 1 and 2 µM drug. These data suggest resistance to ATO and resistance to melphalan are controlled by overlapping pathways, whereas an as of yet uninvestigated pathway plays a role in doxorubicin and ATO cross-resistance.

## Discussion

In an effort to better understand drug action and resistance mechanisms in multiple myeloma, we attempted to generate arsenic resistant myeloma cell lines. Unfortunately after multiple attempts in 4 different backgrounds we could only generate a single line, 8226-ATOR05. This suggests that arsenic targets critical pathways for myeloma cell survival. Therefore understanding these pathways could provide insight into how to better target the disease. Acquired resistant lines have been useful for determining both drug targets through loss of function changes as well as compensatory mechanisms of resistance. Our data suggest there are two pathways involved in arsenic resistance in 8226/S-ATOR05 cells and the importance of each pathway is predicated on ATO concentration. At lower, clinically relevant, concentrations the primary mechanism responsible for resistance appears to be alterations in the expression of two Bcl-2 family members, Bfl-1 and Noxa, whereas elevated levels of intracellular GSH are required for resistance to super-clinical doses.

The role of GSH in drug metabolism, and specifically arsenic metabolism is well documented. Based on previous studies it is not surprising that we found an elevated level of intracellular GSH in our ATO resistant cell line [29]. However what is intriguing is the apparent dose dependent role. GSH is thought to be important for maintaining the intracellular redox status in response to ATO as well as direct binding and export of the arsenic molecule. Our laboratory has previously shown that the production of ROS in response to ATO treatment is not important for the induction of apoptosis, suggesting the primary role GSH plays in 8226/S-ATOR05 is one of detoxification rather than antioxidant defense [25]. Consistent with these findings, we observed no changes in the Nrf2 anti-oxidant pathway associated with acquired resistance to ATO. Therefore it is understandable that the requirement for GSH is much higher when the intracellular level of arsenic is high. The cell would need to be highly efficient at neutralizing and removing excess arsenic so that the survival pathways are not overwhelmed. This is further supported by the fact that GSH levels return to baseline rapidly upon the removal of arsenic selection. In our previous studies of the myeloma response to arsenic trioxide we found that numerous genes involved in de novo GSH synthesis as well as the GSH salvage pathway were up-regulated within 24 h of ATO treatment [25]. Surprisingly none of these genes were found to be up-regulated in ATOR05 cells. These genes were all associated with the Nrf2 response which is not elevated during acquired resistance, therefore this finding is not surprising. However one gene in the *de novo* pathway is modestly up-regulated and returns toward baseline expression once the cells were removed from arsenic selection. While not the rate-limiting step in GSH synthesis, GSS is required to link glycine to the γ-glutamyl-cysteine to complete the synthesis [31]. Both the magnitude and temporal nature of the change in GSS expression are consistent with the changes observed in GSH levels and would be the most likely explanation for the increase in GSH levels associated with acquisition of ATO resistance.

Bcl-2 family proteins are important regulators of the intrinsic apoptotic cascade [32]. Alterations in the regulation or expression of one or more of these proteins are a hallmark of many cancers and often contribute to chemo-resistance [33]. Here we report changes in the expression of two Bcl-2 family members, Bfl-1 and Noxa, in our ATO resistant cell line. Bfl-1 is an anti-apoptotic protein whose up-regulation contributes to chemo-resistance in B-cell chronic lymphocytic leukemia [34] as well as acquired resistance to ABT-737 in diffuse large B-cell lymphoma cell lines [35]. The up-regulation of Bfl-1 in 8226/S-ATOR05 is associated with resistance to ATO at low concentrations and may play a role with the increase in GSH to promote resistance at super-clinical doses. Bfl-1 is an attractive candidate for an ATO resistance gene as it can effectively bind to the pro-apoptotic BH3-only activator protein Bim and is not inhibited by the BH3-sensitizers Noxa or BMF [36]. We have previously shown that these three BH3-only proteins are required for ATO-induced apoptosis in myeloma cell lines [21]. Interestingly Bfl-1 is the only known anti-apoptotic Bcl-2 family member that is not inhibited by BMF and thus would allow cells to overcome BMF induction by ATO. The mechanism of Bfl-1 induction during acquisition of ATO resistance is not addressed in the current study however it is interesting to note that this is an established target of NF-κB signaling [37]. However other NF-κB targets including Bcl-x_L_, cIAP2, and IκB are not changed in these cells. Additionally none of the NF-κB genes display altered expression in ATO-R05 cells (unpublished observations). Finally bortezomib sensitivity is not altered in these cells, therefore the mechanism does not appear to be related to NF-κB regulation and warrants further investigation. Unfortunately silencing of Bfl-1 in ATOR05 was only modestly effective making it very difficult to directly test the role of Bfl-1 in this model (not shown). While the levels of Bfl-1 could be reduced by over 50% at the mRNA level, the cells still express much higher levels than the parental or CR cells making the results of these studies difficult to interpret. Regardless the up-regulation of anti-apoptotic Bcl-2 proteins in myeloma cells can influence ATO-induced apoptosis and up-regulation of Bfl-1 would be consistent with these findings [9].

Noxa is a pro-apoptotic BH3-only member of the Bcl-2 family that inhibits Mcl-1 [32] [36]. We have previously shown that Noxa is up-regulated in response to ATO in multiple myeloma cells and that it plays a pivotal role in the initiation of apoptosis in response to arsenic [21] [24]. It is not surprising then that the loss of this induction would contribute to ATO resistance. However the apparent inverse relationship between Noxa and GSH is interesting. We have shown that when myeloma cells are treated with ATO, Noxa is induced, and when cells are treated with ATO and NAC (to enhance GSH production) Noxa induction is decreased. However when cells are treated with ATO and BSO (to deplete GSH) Noxa induction is significantly increased [21]. It is unlikely that Noxa induction is dependent upon ROS production, as treating cells with ATO and then blocking ROS production with BHA does not block Noxa up-regulation. Additionally RPMI-8226 expresses a mutant form of p53 therefore the induction is independent of this pathway [38]. A third BH3 protein which is induced by ATO in myeloma is Puma. However our previous studies indicated that Puma induction was not required for ATO-induced apoptosis [21]. In our current studies Puma baseline expression is lower in the ATO-resistant cells, however consistent with a lack of a role for Puma in ATO-induced death, Puma induction remains intact in ATOR05.

In addition to arsenic resistance, we have shown that 8226/S-ATOR05 cells are also resistant to melphalan and doxorubicin but not to other agents including the organic arsenical darinaparsin. This latter finding is consistent with our previous studies that demonstrated that intracellular GSH levels were not as important in regulating darinaparsin-induced death as they are for ATO [24]. Melphalan and doxorubicin both function by damaging DNA, acting as either an alkylating or intercalating agent, respectively, ultimately inhibiting replication and inducing apoptosis in rapidly dividing cancer cells. While these mechanisms of action differ from that of ATO our data suggest that either one or both of the pathways responsible for arsenic resistance in 8226/S-ATOR05 cells are also involved in loss of sensitivity to Mel and Dox. Further examination of the data suggest that perhaps elevated intracellular GSH is important for resistance to melphalan as cells once again become sensitive to melphalan as GSH levels decrease, similar to the pattern see with ATO. This is consistent with the selection of melphalan resistance in the RPMI-8226 background. The LR5 mutant was selected for melphalan resistance and it also displays an increase in GSH levels [39]–[40]. The mechanism of resistance to doxorubicin appears to be at least partially distinct from melphalan and even ATO as it is stable for at least 50 days following ATO withdrawal. This is different than what was observed when RPMI-8226 cells were selected for doxorubicin resistance. The Dox40 mutant is primarily resistant to doxorubicin because of up-regulation of p-glycoprotein and Bcl-x_L_ and are not resistant to ATO [41]–[42] [9]. Despite having activity, the future use of ATO in the treatment of myeloma is not clear, however it may prove to be an important tool for the study of resistance mechanisms to drugs that remain as workhorses in the treatment of this disease.

Finally, our data point to a potential problem with data interpretation using acquired resistant lines to determine mechanism of action or resistance. While our line was generated using clinically relevant arsenic levels (1 µM), it became resistant to concentrations eight times higher. Our data suggest that the important aspects of resistance to clinically achievable concentrations may differ from those required for “maximal” resistance and caution should be taken in determining the appropriate mechanisms of resistance in these lines.
